# Expression of SUR1 isoforms in the brain and heart after ischemia/reperfusion

**DOI:** 10.3389/fnmol.2025.1536409

**Published:** 2025-04-17

**Authors:** Iván Alquisiras-Burgos, Irlanda Peralta-Arrieta, Mónica Espinoza-Rojo, Alejandro Salazar-Salgado, Iván Antonino-Olguín, Alicia Sánchez-Mendoza, María Sánchez-Aguilar, Martha-Eugenia Ruiz-Tachiquín, Hilda-Alicia Valdez-Salazar, Alma Ortiz-Plata, Javier Franco-Pérez, Arturo Hernández-Cruz, Penélope Aguilera

**Affiliations:** ^1^Laboratorio de Patología Vascular Cerebral, Instituto Nacional de Neurología y Neurocirugía Manuel Velasco Suárez, Ciudad de México, Mexico; ^2^Laboratorio de Transducción de Señales, Instituto Nacional de Enfermedades Respiratorias Ismael Cosío Villegas, Ciudad de México, Mexico; ^3^Laboratorio de Biología Molecular y Genómica, Facultad de Ciencias Químico-Biológicas, Universidad Autónoma de Guerrero, Chilpancingo de los Bravo, Mexico; ^4^Departamento de Neuropatología Molecular, Instituto de Fisiología Celular, Universidad Nacional Autónoma de México, Ciudad de México, Mexico; ^5^Departamento de Farmacología, Instituto Nacional de Cardiología Ignacio Chávez, Ciudad de México, Mexico; ^6^Unidad de Investigación Médica en Enfermedades Oncológicas, Centro Médico Nacional Siglo XXI, Instituto Mexicano del Seguro Social, Ciudad de México, Mexico; ^7^Unidad de Investigación Médica en Enfermedades Infecciosas y Parasitarias, Centro Médico Nacional Siglo XXI, Instituto Mexicano del Seguro Social, Ciudad de México, Mexico; ^8^Laboratorio de Patología Experimental, Instituto Nacional de Neurología y Neurocirugía Manuel Velasco Suárez, Ciudad de México, Mexico

**Keywords:** sulphonylurea 1 receptor, SUR1, brain ischemia, heart ischemia, Abcc8

## Abstract

The sulfonylurea receptor 1 (SUR1) has been classified as a member of the adenosine triphosphate (ATP)-binding cassette (ABC) transporter superfamily. SUR1, unlike the classic ABC transporters, assembles with Kir6.2, forming K_ATP_ channels to regulate the flux of potassium ions. In the central nervous system, SUR1 is weakly expressed in some brain regions but is induced by pathological conditions in the different cell types of the neurovascular unit. Therefore, we first analyzed the expression of SUR1 in various rat tissues and brain regions to identify SUR1 isoforms and their mRNA exon composition under physiological conditions. Later, we focused on the SUR1 expression in the brain and heart after ischemia/reperfusion. We observed two SUR1 isoforms (170 and 60–75 kDa) abundantly expressed in most rat tissues, except for the testis and brain, where basal expression of these isoforms was relatively low and exhibit a band of 100 kDa. Every exons coding for the functional domains of SUR1 mRNA were amplified from the tissues and brain regions analyzed. Results from *in vitro* and *in vivo* experiments indicated that SUR1 isoforms previously identified (170 and 60–75 kDa) were dramatically overexpressed in the brain after middle cerebral artery occlusion followed by reperfusion. In contrast, myocardial infarction followed by reperfusion significantly reduced SUR1 isoform expression in the heart. This study demonstrates the expression of at least two SUR1 isoforms in various tissues and suggests that ischemic processes may differentially regulate SUR1 expression depending on the tissue injured.

## 1 Introduction

**Figure 1:**
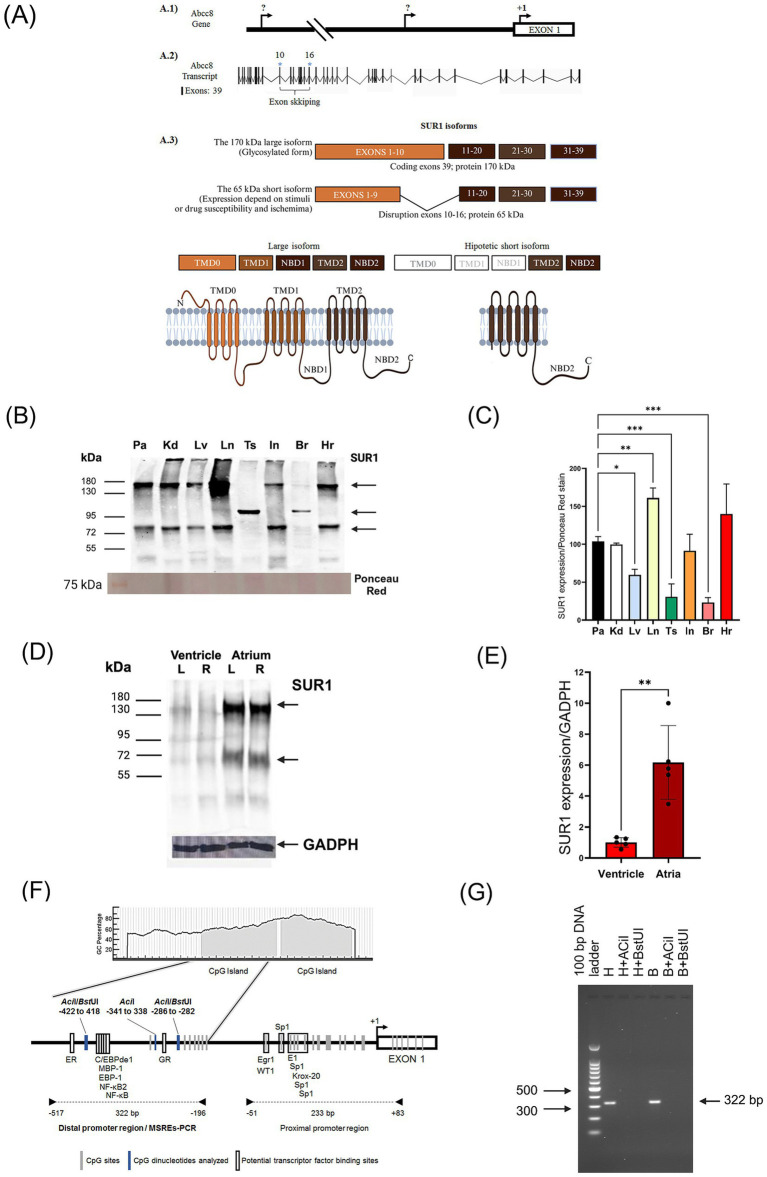
**(A)** Schematic representation of SUR1 isoforms and their potential generation mechanisms. (A.1) It has been proposed that the short 65 kDa isoform could originate from alternative transcription start sites in the Abcc8 gene. (A.2) Another possibility is alternative splicing, which removes exons 10 to 16 (marked with a blue asterisk), resulting in the loss of the TMD0, TMD1, and NBD1 domains, a mechanism similar to that described for the 63 kDa SUR2 isoform (Pu et al., 2008; Ye et al., 2009). (A.3) Schematic representation of the full-length SUR1 protein domains: TMD0 (Transmembrane Domain 0), TMD1 (ABC-transporter Transmembrane Domain 1), NBD1 (Nucleotide-Binding Domain 1), TMD2 (Transmembrane Domain 2) and NBD2 (Nucleotide-Binding Domain 2). Additionally, a hypothetical topological model of the short 65 kDa isoform is shown, which could retain the carboxyl-terminal domain according to our previous research (Alquisiras-Burgos et al., 2022). **(B)** Expression of SUR1 isoforms (170 and 60–75 kDa) in rat tissues (Pa: pancreas, Kd: kidney, Lv: liver, Ln: lung, Ts: testis, In: intestine, Br: brain, Hr: heart). **(C)** Quantification of the 170 kDa band normalized to Ponceau red stain. **(D)** SUR1 isoforms (170 and 60–75 kDa) were expressed in heart chambers (L: left; R: right). **(E)** Quantification of the 170 kDa SUR1 isoform in the ventricle and atrium. **(F)** Prediction of the CpG islands in the Abcc8 gene promoter of the Rattus norvegius (NC_051336.1) using the MethPrimer v1.1 beta. Oligonucleotide sequences designed for polymerase chain reaction (PCR) amplification of the distal and proximal regions are indicated (arrows). The methylation-sensitive restriction enzymes (MSRE) sites are shown in bold. Potential transcription factor binding sites are represented as white boxes (TRANSFAC 4.0 sites: http://gene-regulation.com/pub/programs/alibaba2/index.html). **(G)** Methylation in the brain and heart was analyzed with the MSRE-PCR system. PCR products were separated by electrophoresis. No methylation was detected in the heart (H + ACiI, H + BstUI) and brain (B + ACiI, B + BstUI). Results of three or four independent experiments (mean ± SEM) *: *p* < 0.05, **: *p* < 0.01, ***: *p* < 0.001.

The sulfonylurea receptor (SUR) has been classified as a member of the adenosine triphosphate (ATP)-binding cassette (ABC) transporter superfamily. The ABC superfamily is a large group of membrane proteins widely distributed in all phyla and associated with various functions, such as the transport of ions and molecules, drug resistance, toxin secretion, and lipid trafficking (Liu, 2019; Ogasawara et al., 2020). The mammalian ABC superfamily has been classified into different subfamilies or types based on gene structure, domain order, nucleotide-binding domain sequence homology, and transmembrane domain fold (Thomas et al., 2020). Therefore, the ABCC subfamily contains thirteen members with sizes from 1,325 to 1,585 amino acids, including mainly multidrug resistance proteins (MRPs) and two sulfonylurea receptors SUR1 (ABCC8) and SUR2 (*Abcc9*) (Alam and Locher, 2023). The human *Abcc8* gene encoding the SUR1 protein consists of 39 exons distributed in 84,397 bp located on chromosome 11p15.1. The 5′ untranslated region of the SUR1 transcripts is identical in mouse *β*-cells and the brain, suggesting that different cell types share the same transcription start site. Thus, the open reading frame of SUR1 cDNA encodes a protein of 1,581 amino acids with a mass of 176,992 Da, although this mass may vary slightly due to post-translational modifications (Genbank NG_008867.1; GC11M017392; Stelzer et al., 2016). SUR1 protein contains three hydrophobic domains (TMD0, TMD1, and TMD2) linked by the cytosolic linker region (L0) and two hydrophilic nucleotide-binding domains (NBD1 and NBD2). Transmembrane topological analysis indicates the presence of 17 transmembrane helices disposed in a 5 + 6 + 6 array in the TMD0, TMD1, and TMD2 domains of SUR1 (Conti et al., 2001; Driggers and Shyng, 2023). The full-length SUR1 protein is around 170 kDa, while smaller isoforms (140, 100, and 65 kDa) may result from alternative splicing or proteolytic processing (Figure 1A). Although some studies have detected the shorter isoforms, the precise roles remain unclear, suggesting they may have different regulatory properties, affecting channel conductance, trafficking, or interaction with other proteins (Alquisiras-Burgos et al., 2022).

SUR1 is a regulatory subunit assembled with Kir6.2, an inward rectifier potassium channel, to form K_ATP_ channels. Thus, the functional K_ATP_ channel is a hetero-octamer, composed of four central Kir6.2 subunits surrounded by four SUR1 subunits, and all of these structural elements form, operate, and regulate the channel complex (Driggers and Shyng, 2023; Patton et al., 2024). Electrophysiological studies have shown that K_ATP_ channels are inhibited by ATP binding to Kir6.2 while Mg^2+^-complexed ADP interacting with SUR1 activates the channel, thus increasing the probability of an open channel conformation (Craig et al., 2008; Usher et al., 2020). Early genetic studies linked mutations in the ABCC8 gene to familial hyperinsulinemic hypoglycemia of infancy, an autosomal recessive disorder characterized by unregulated insulin secretion (Thomas et al., 1995). Later, Patch et al. (2007) reported that SUR1 mutations could cause transient neonatal diabetes mellitus and permanent diabetes beyond the neonatal period. In pancreatic *β*-cells, sulfonylurea drugs facilitate insulin secretion by antagonizing the bound Mg^2+^-ADP at NBD2 in SUR1 and inactivating the K_ATP_ channel (Ueda et al., 1999). These drugs potently lower glucose levels through this mechanism and represent a therapeutic option in treating type 2 diabetes mellitus (Tomlinson et al., 2022).

Some studies have analyzed SUR1 expression across various tissue types of the rat and described that SUR1 transcripts are abundant in the pancreas and brain but are also present in the heart, skeletal muscle, lung, liver, kidney, and stomach (Hernández-Sánchez et al., 1997; Shi et al., 2005; Alquisiras-Burgos et al., 2022). As the regulatory subunit of the K_ATP_ channel, it is considered that SUR1 plays essential roles in a wide variety of physiological processes, including hormonal function by controlling insulin secretion in pancreatic *β*-cells, neuronal and cardiac modulation by contributing to membrane potential and cellular excitability as well as regulating vascular tone in smooth muscles (Proks et al., 2002; Foster and Coetzee, 2016; Bal et al., 2018). Interestingly, emerging research has evidenced the role of SUR1 in diverse pathological conditions. Elrod et al. (2008) observed that SUR1-null mice were markedly protected against the damage induced by myocardial ischemia/reperfusion and suggested a regulatory mechanism of the SUR1 subunit in cardiovascular homeostasis. Remarkably, elevated SUR1 expression has been detected in preclinical models and patients after brain ischemia/reperfusion, playing a role in cerebral edema and necrotic cell death (Mehta et al., 2013; Alquisiras-Burgos et al., 2020). These results show the complexity of SUR1 in the function of K_ATP_ channels; however, most reports have focused on detecting the transcript and analyzing its function by electrophysiology. Therefore, under physiological conditions, we first examined the protein expression of SUR1 in different rat tissues and brain regions to identify SUR1 isoforms; then, we detected SUR1 mRNA exon composition. Finally, we evaluated SUR1 expression *in vivo* and *in vitro* after ischemia/reperfusion and observed that this process may differentially regulate SUR1 expression depending on the tissue injured by ischemia.

## 2 Methods

### 2.1 Animals

Thirty male Wistar rats (260–320 g) were used for this protocol. Rats were maintained under controlled conditions of temperature (22 ± 2° C), humidity (52%), and light (12:12 light–dark cycle). Food and water were available *ad libitum*. All animal experiments were approved (no. 92/20) by the Institutional Committee for Care and Use of Laboratory Animals (CICUAL-INNN). Treatments and euthanasia were carried out according to Mexican Official Norms for the production, care, and use of laboratory animals (NOM-062-Z00-1999).

### 2.2 Cell culture

Human brain endothelial cells (HBEC-5i) and human cardiomyocytes derived from pluripotent stem cells (hPSC-CMs) obtained as reported by Lin and Zou (2020) and Wang et al. (2021) were maintained with culture medium under normoxic conditions (37° C, 5% CO_2_, and 18% O_2_) in an incubator INCO108med (Memmert, Germany). For oxygen and glucose deprivation (OGD), cells were incubated with a glucose-free medium under hypoxic conditions (1% O_2_, 94% N_2_, and 5% CO_2_) at 37° C for 2 h. After OGD, the glucose-containing medium was restored and the cells were incubated for 24 h (recovery period) under normoxic conditions. Cells from the control group were maintained in a glucose-containing medium and normoxic conditions throughout the experiment.

### 2.3 Reverse transcription polymerase chain reaction (RT-PCR), endpoint PCR, and methylation-sensitive restriction enzymes

SUR1 exons expression in tissues and brain regions was evaluated using 5 μg RNA extracted with TRIzol^™^ reagent followed by RT-PCR performed using the SuperScript^™^ III One-Step RT-PCR System (Cat. 125740 Invitrogen). Reactions were executed in triplicate following the manufacturer’s guidelines. Seven pairs of primers in exons coding for different domains of the complete protein were used to identify truncated regions (Supplementary Table 1).

Methylation in the brain and heart was analyzed using the methylation-sensitive restriction enzymes PCR method. DNA was extracted from rat tissues using the DNeasy^®^ Blood & Amp Tissue Kit (Qiagen, Netherlands). DNA quality was determined using a Multiplex PCR kit (Qiagen, Netherlands) (Valdez-Salazar et al., 2020). Then, DNA was restricted with Aci1 and BstU1, and the distal region of the promoter (517 to −196 bp relative to the transcription start site) amplified. The endpoint PCR reaction mixture contained 1X Go Taq Flexi Buffer 5X Colorless (Cat. M890A Promega, United States), GoTaq G2 Hot Start Polymerase (Cat. M7408B Promega, United States), 250 uM deoxyribonucleotides, 1.5 mM MgCl_2_, 0.2 μM each oligonucleotide (forward 5’ ATCACCACAAGGGAAGAGAGC -3′ and reverse 5′-GGTGTCTGAGTGGGAGTCGT-3), 10 ng DNA, and nuclease-free water. Amplification was performed in a MasterCycler GSX1 (Eppendorf, Germany) as follows: 95°C for 5 min, followed by 35 cycles of 94°C for 30 s, 59°C for 30 s, 72°C for 45 s, 72°C for 10 min, and a final stage at 4^o^ C indefinitely.

PCR products were separated by electrophoresis on a 2% agarose gel with a 100 bp DNA ladder (Promega, United States), stained with 1X GelRed^™^ (Biotium, Inc., United States), and visualized under an ultraviolet light transilluminator and imaging system (Syngene, United States).

### 2.4 Western blot

Tissues (pancreas, kidney, liver, lung, testis, intestine, brain, and heart) were homogenized in RIPA buffer containing a cocktail of protease inhibitors (Sigma Aldrich, United States) with the Sonics vibra cell at 60% amplitude for 10 s. For harder tissues, the process was repeated 2 to 3 times. For the heart, a Polytron homogenizer PT-MR2100, Kinematica AG (Switzerland) was used in Figure 1A. The homogenates were centrifuged, and the protein concentration was measured using the BCA method. Proteins (50 μg) were separated by SDS-PAGE (8%), transferred onto nitrocellulose membranes (Bio-Rad, United States), and blocked with 5% nonfat dry milk diluted in TBST. Following, membranes were incubated overnight at 4°C with anti-SUR1 sc-293436 (1:100). Clonality: monoclonal clone number 3G5. Isotype: IgG2a. Predicted molecular weight: 180 kDa; Immunogen: aa 611–710. Specificity: reacts with rat and human. Positive control for WB: human recombinant SUR1 fusion protein. Santa Cruz Biotechnology, United States. Or anti-SUR1 ab134292 (1:100). Clonality: monoclonal clone number N289/16. Isotype: IgG1. Predicted molecular weight: 177 kDa; Immunogen: fusion protein corresponding to Rat SUR1 aa 1,500 to the C-terminus. Database link: Q09429. Specificity: reacts with rat and human and does not cross react with SUR2B. Positive control for WB: rat brain membrane lysate. Abcam, UK. After washing with TBST, membranes were incubated with anti-mouse IgG HRP conjugated (1:3000, JIR-115-035-062, Jackson ImmunoResearch, United States). The visualization of protein bands was performed by chemiluminescence using Luminata Forte (Millipore, United States) and an imaging system (Fusion Solo S, France). After stripping, membranes were incubated with anti-*α* tubulin (1:1000, T9026, Sigma-Aldrich, United States) for the brain or anti-GAPDH (1:1000, 14C10, Cell Signaling, United States) for the heart as loading proteins reference. A Ponceau staining was used as a loading reference when different tissues were analyzed.

### 2.5 Immunofluorescence

HBEC-5i and hPSC-CMs cells were seeded on 12 mm round coverslips at 10,000 cells/coverslip density. After OGD, cells were fixed with cold methanol, washed, and kept in cold PBS until use. Cells were blocked with 10% goat serum and then incubated with primary antibodies against SUR1 (1:200 dilution, SC-293436, Santa Cruz Biotechnology, United States) or NKX 2.5 (1:100 dilution, PA5-4943, ThermoFisher Scientific, United States) or GAPDH (1:600, SC-25778, Santa Cruz Biotechnology, United States) at 4°C overnight. After washing with PBS, cells were incubated with antibodies anti-IgG conjugated with DyLight^™^ 488 (JIR-315-485-044) or Alexa Fluor^®^ 594 (JIR-711-585-152) for 2 h. Then, cells were incubated with DAPI. Images were acquired with an inverted microscope (Olympus 1×71, Olympus Corporation, United States).

### 2.6 Monitoring of intracellular Na^+^

As previously described (Alquisiras-Burgos et al., 2024), cells (HBEC-5i) were seeded on 24 mm diameter round coverslips (20,000 cells/coverslip), washed with KHB solution and immediately incubated at 37°C, 5% CO_2_, with a fluorescent probe (CoronNa Green, 5 μM) (Sigma-Aldrich, United States), diluted in KHB + glucose (10 mM) for 45 min. Then, cells were transferred to a perfusion chamber on the plate of the upright microscope with a 20×, 0.95 NA water-immersion objective (Eclipse Ni. Nikon, Japan). The Na^+^ concentration changes were evaluated after the emission of illumination pulses at 488 nm (50- to 100-ms exposure) with a disk-spinning scanning confocal system (CSU-X1, Solamere Technology Group, United States). Images were acquired with a cooled digital camera (Prime 95B, Photometrics, United States), and time-lapse recordings (approximately 1,600 images) were acquired at 500 ms intervals. A physiological solution followed by Diazoxide (100 μM) was applied for 5 min. The analysis was performed with Image J software 1.8.0. Fluorescence was reported in arbitrary units (a.u).

### 2.7 Intracellular calcium recordings

hPSC-CMs were seeded on 18 mm round glass coverslips and incubated with 2.5 μM of the fluorescent calcium indicator Fluo-4 AM (Sigma-Aldrich, United States) for 30 min at 37°C. Coverslips were transferred to a perfusion chamber on the plate of an upright Nikon Eclipse 80i microscope equipped with a 20×, 0.95 NA water-immersion objective. Fluo-4 AM was excited at 488 nm with a solid-state Coherent Obis laser, coupled to a CSUXM1Yokogawa spin-disk confocal scan head. Emitted fluorescence was band-passed (460/50), and images were collected with a sCMOS camera (Prime 95B, Photometrics). The acquisition and illumination were controlled with Micro-manager software. Time-lapse recordings (approximately 1,600 images) were acquired at 175 ms intervals and analyzed with Fiji (Image J) software. A physiological solution was perfused first into the chamber, followed by 5 min of Diazoxide (100 μM) containing solution. Fluorescence data is reported in arbitrary units (a. u).

### 2.8 Middle cerebral artery occlusion (MCAO)

MCAO was performed according to the technique previously described by Longa et al. (1989). Rats were anesthetized with 3% isofluorane (Pisa, México) and placed under a surgical microscope (Olympus Optical, Japan). A neck incision was made to visualize the common carotid artery (CCA), the internal carotid artery (ICA), and the external carotid artery (ECA). ECA was ligated distally with a 6–0 silk suture; ICA was also ligated but right at CCA bifurcation to stop blood flow. A small incision was made in the CCA to insert a 3–0 nylon monofilament (~17 mm) directed toward the ICA. In this way, the monofilament reached and occluded the middle cerebral artery. The occlusion was maintained for 2 h. After, the animals were re-anesthetized, and the filament was removed to allow reperfusion. Control rats underwent the same surgical procedure except the MCAO. Rats were sacrificed after 24 h of reperfusion.

### 2.9 Myocardial infarction

Rats were deeply anesthetized, and oro-tracheal intubation was performed, allowing artificial ventilation (RF: 70′, TV: 8 L/min) (Ugo Basile, Italy). A skin incision was made between the third and fourth ribs, and the left anterior descending coronary artery was ligated with a 6–0 polypropylene suture and a polypropylene tubing (PE-10) for 30 min. Then, the tubing was removed, allowing myocardium reperfusion (Oidor-Chan et al., 2016). The thoracic cavity was sutured, and negative pressure was restored. Rats were administered with a single dose of analgesic (tramadol 5 mg/kg) and allowed to recover from anesthesia. Control rats underwent the same procedure but without coronary artery ligation. Twenty-four hours later, subjects from both groups were anesthetized and sacrificed, and cardiac hearth samples were obtained.

### 2.10 Statistical analysis

Statistical analyses were performed using GraphPad Prism 9.0. We used a t-*test* to compare the results from the experiments of monitoring intracellular Na^+^ and intracellular calcium recordings. Other experiments were compared with one-way ANOVA; the Tukey test was used to compare the means of three or more groups. A value of *p* < 0.05% was considered as significant.

## 3 Results

### 3.1 SUR1 is expressed in most of the rat tissues

We used two different anti-SUR1 antibodies from Santa Cruz Biotechnology (sc-293436) and Abcam (ab134292) for the Western blot analysis. Similar results were obtained with both antibodies; therefore, we used the antibody sc-293436 for all the experiments. We detected that two SUR1 isoforms (170 and 60–75 kDa) are abundantly expressed in most rat tissues, except for the brain and testis, where basal expression of these isoforms is low. We also detected a band of 100 kDa in the testis and brain (Figures 1B,C). The SUR1 protein displays strong expression in cardiac chambers, with a higher expression level in the atrium than in the ventricles (Figures 1D,E). We hypothesized that the significant difference in SUR1 expression in the heart compared to the brain could be associated with epigenetic regulation. Accordingly, using the Meth Primer program (Li and Dahiya, 2002), we identified two CpG islands in the *Abcc8* gene promoter, which possess binding sites for multiple transcription factors (Figure 1F). Then, we compared the methylation of the distal region of the *Abcc8* promoter in the brain and heart through the methylation-sensitive restriction enzymes method; nonetheless, no amplification product was obtained (Figure 1G). This result demonstrates that the AciI and BstUI sites at the analyzed region were not methylated.

### 3.2 The mRNA of SUR1 contains the exons associated with the protein domains

**Figure 2:**
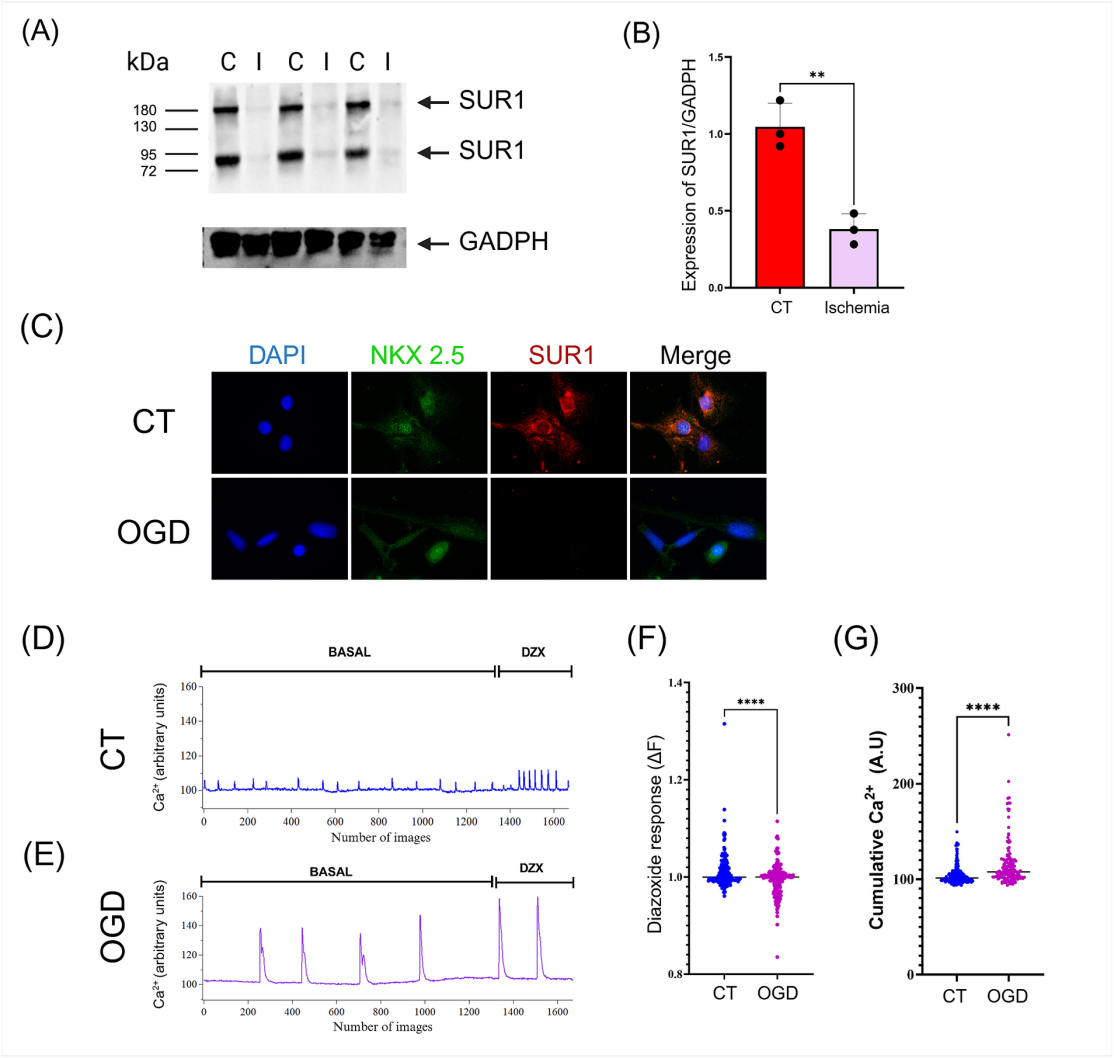
**(A)** The expression of SUR1 isoforms in the heart was significantly reduced after myocardial infarction/reperfusion compared to control (I: Ischemia; C: control). **(B)** Quantification of SUR1 (170 kDa) levels normalized to GADPH expression. **(C)** *In vitro*, oxygen and glucose deprivation (OGD)/recovery induced downregulation of SUR1 in human cardiomyocytes (hPSC-CMs). **(D-E)** hPSC-CMs cultures were subjected to control conditions and OGD/recovery and then stimulated with Diazoxide (DZX, 100 μM). Calcium recordings were performed using a fluorescent probe Fluo-4. **(F)** The response to Diazoxide was calculated using the fluorescence change equation (∆F = Ff − Fi). **(G)** The activity of each cell was quantified by measuring the accumulated calcium under basal conditions. Results of three or four independent experiments (mean ± SEM) *: *p* < 0.05, **: *p* < 0.01, ***: *p* < 0.001, ****: *p* < 0.0001.

We used seven pairs of primers situated in exons coding for the functional domains of the complete protein to identify truncated regions and to determine whether the SUR1 isoforms found in rat tissues originated from mRNAs of different sizes (Figure 2A). Our results showed that SUR1 mRNAs expressed in all the tissues analyzed contain each of the selected exons, except for exons 1 and 2 (E1-E2), which did not amplify in the pancreas, E17-E18 in the liver, and E35-E36 in the heart and pancreas (Figure 2B). We also found that all exons were amplified in each brain region analyzed (cortex, hippocampus, hypothalamus, cerebellum, and olfactory bulb) (Figure 2C), even though the expression level of SUR1 protein observed in the brain is relatively low (Figures 1B,C). Additional primers were designed to detect exon 1 because the first pair used did not amplify in any of the tissues tested. Thus, with these additional primers, exon 1 was amplified in all tissues and brain regions analyzed except the pancreas (Figures 2D,E).

### 3.3 MCAO induced up-regulation of SUR1 expression in the brain

**Figure 3:**
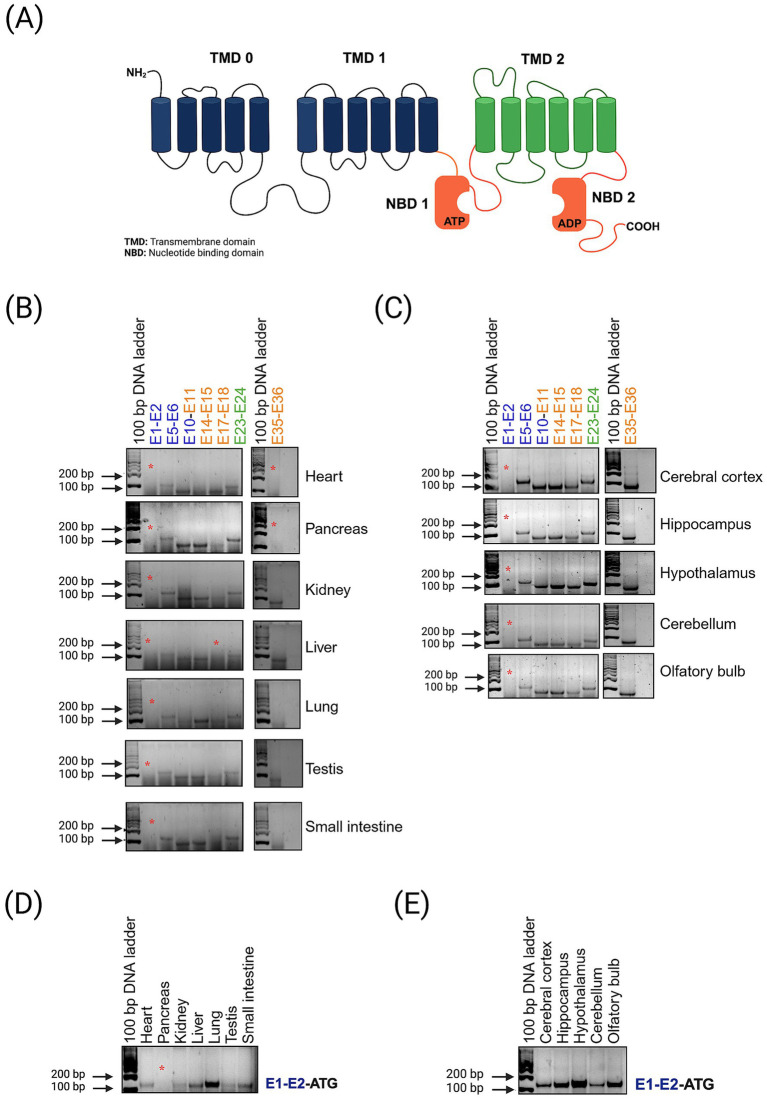
**(A)** Topological structure of the SUR1 protein. **(B,C)** Agarose gel (3%) electrophoresis of PCR products generated by amplifying exons of the *Abcc8* gene in different tissues and brain regions (the sequence of primers is shown in the supplementary table). The asterisk indicates no-amplified PCR product. **(D,E)** A second pair of primers was designed for exon 1 and exon 2.

We induced transient MCAO in the rat for 2 h, followed by 24 h of reperfusion. This experimental model of stroke significantly augmented the level of both SUR1 isoforms in the brain (Figures 3A,B). Similarly, 2 h OGD followed by a 24 h recovery period induced SUR1 expression in the brain micro endothelial cell culture (Figure 3C).

SUR1 binds to the transient receptor potential melastatin 4 channel (TRPM4) and aquaporin 4, forming a complex that regulates the influx of Na^+^ and water (Stokum et al., 2023). Accordingly, we observed that the brain micro endothelial cells exposed to OGD followed by 24 h recovery period rise intracellular Na^+^ compared to control cells (cumulative Na^+^) (Figures 3D–F). Additionally, when these cultures were stimulated with the SUR1 activator (Diazoxide) they showed an augmented content of cellular Na^+^ compared with basal conditions (Figures 3D,E,G).

### 3.4 Myocardial infarction reduces the cardiac expression of SUR1

**Figure 4:**
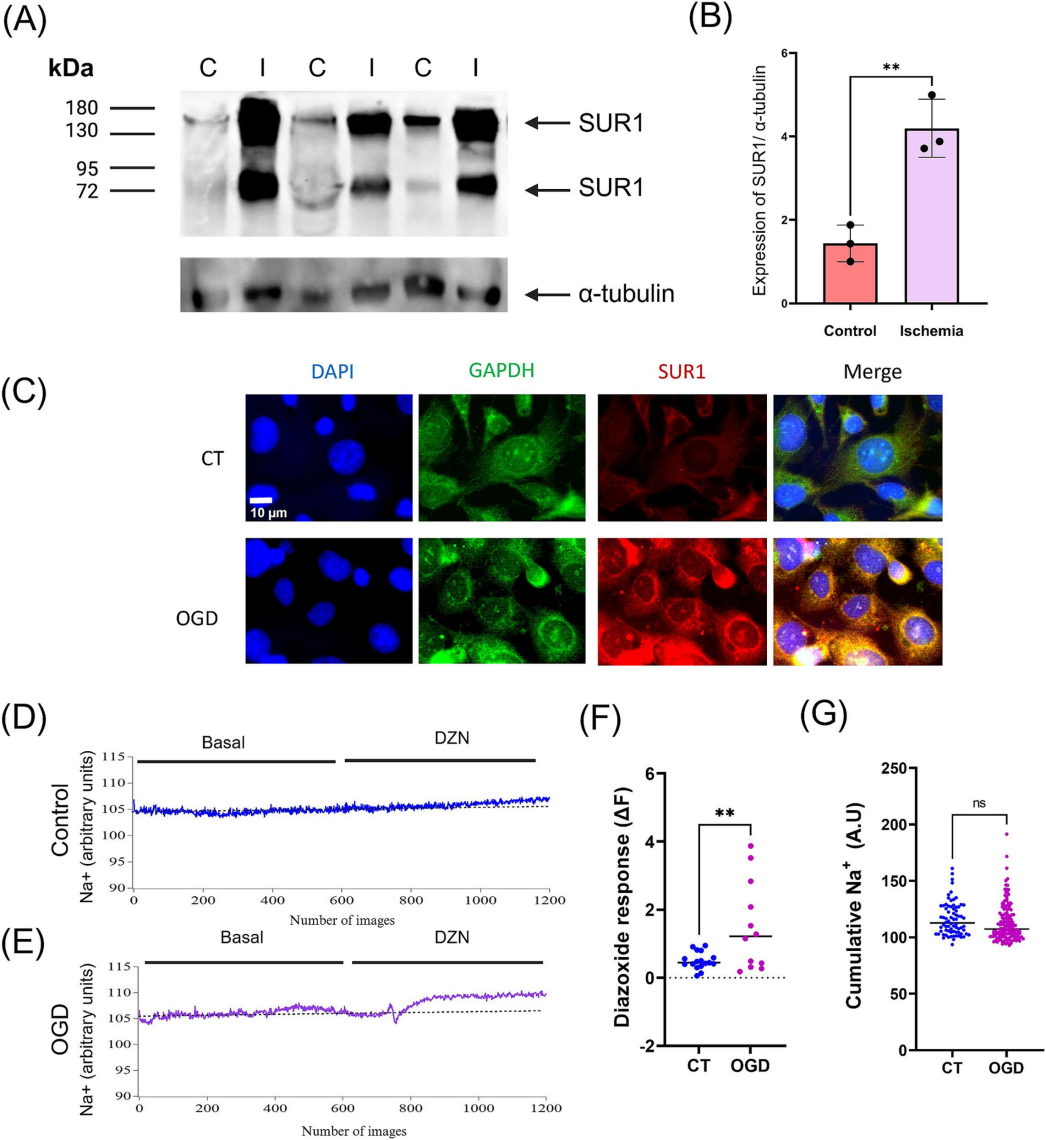
**(A)** Analysis of SUR1 isoforms expression by western blot showed a significant increase in the brain after middle cerebral artery occlusion/reperfusion compared to control (I: Ischemia; C: control). **(B)** Quantification of SUR1 levels normalized to *α*–tubulin expression. **(C)** *In vitro*, oxygen and glucose deprivation (OGD)/recovery induced a downregulation of SUR1 in human brain endothelial cells (HBEC-5i). **(D,E)** HBEC-5i cultures were subjected to control conditions and OGD/recovery; then, they were stimulated with Diazoxide (DZX, 100 μM). Monitoring of intracellular Na^+^ was performed using CoronNa Green. **(F)** The response to Diazoxide was calculated using the fluorescence change equation (∆F = Ff − Fi). **(G)** The activity of each cell was quantified by measuring the accumulated sodium under basal conditions. Results of three or four independent experiments (mean ± SEM) **: *p* < 0.01, ***: *p* < 0.001.

SUR1 was abundantly expressed in the heart under control conditions. To find out if hypoxia changed this situation, we induced myocardial infarction for 30 min followed by 24 h of reperfusion. Surprisingly, we found that SUR1 expression in the heart was significantly reduced after ischemia/reperfusion, and this effect was observed with both SUR1 isoforms (170 and 60–75 kDa) (Figures 4A,B). Similarly, cultured human cardiomyocytes exposed to OGD showed a notable reduction in SUR1 expression levels (Figure 4C).

### 3.5 Intracellular Ca^2+^ imaging

Examining Ca^2+^ influx into the intracellular space with fluorescent Ca^2+^-sensitive dyes can be a surrogate for electrophysiological recording of action potential waveforms and firing rate in cardiomyocytes to test for the possible effects of compounds on intracellular Ca^2+^ homeostasis and beating patterns. Under control conditions, hPSC-CMs show spontaneous, regular Ca^2+^ transients. Contractility, action potential generation, and Ca^2+^ transients are common findings in studies with hPSC-CMs because these cells remain immature in culture and resemble heart cells of mid-gestation human fetuses (Veerman et al., 2015).

We evaluated the frequency and amplitude of spontaneous Ca^2+^ transients generated by human cardiac myocytes. After exposure to ODG, cardiomyocytes showed a decrease in the frequency of spontaneous Ca^2+^ transients. Upon incubation with the SUR1 activator (Diazoxide) Ca^2+^ signals increased in frequency and amplitude. These changes in the Ca^2+^ signalling pattern elevate the integral of the intracellular Ca^2+^ fluctuations (cumulative Ca^2+^) in OGD-treated cardiomyocytes more than in the control, untreated ones (Figures 4D–G).

## 4 Discussion

SUR1 is a protein widely expressed in different tissues and cell types; however, most reports have focused on the study of its activity and analysis of the protein with an estimated molecular weight of 170 kDa (Hernández-Sánchez et al., 1997; Shi et al., 2005; Burke et al., 2008). Here, we described the expression of two SUR1 isoforms (170 and 60–75 kDa) with a relative abundance ranked in the following decreasing order: lung>heart>pancreas>kidney>intestine>liver>testis>brain. The heavier protein coincides with the 170 kDa SUR1 complete isoform previously described, while the shorter protein could correspond to an uncharacterized isoform of ≈ 60–75 kDa (Alquisiras-Burgos et al., 2022). Proteins with different molecular weights (140, 100, and 65 kDa) have been detected with antibodies and radioligands in diverse tissues. However, it has not been defined if these proteins are associated with SUR1 isoforms. Interestingly, even the transgenic mouse overexpressing SUR1 in the heart showed increased expression of the three different bands (Alquisiras-Burgos et al., 2022).

Although they are not yet fully identified, the short isoforms are suggested to correspond to SUR subunits of mitochondrial K_ATP_ channels (Grover and Garlid, 2000; Hambrock et al., 2002). However, short isoforms (i.e., SUR2) are also associated with long subunits (Pu et al., 2008). In agreement with the Western blot results, the PCR data evidenced the expression of SUR1 in all the tissues analyzed; SUR1 mRNAs contained each of the selected exons corresponding to the different transmembrane and nucleotide-binding domains.

We found that SUR1 protein displays strong expression in all cardiac chambers, with a higher expression level in the atrium. According to previous reports, the SUR1 transcripts are expressed 7–fold more in the atria than in the ventricles. Moreover, mRNA expression patterns of Kir6.2 and SUR1 are higher in the atrium, while Kir6.1 and SUR2 are higher in the ventricle (Specterman et al., 2023). These differential expression levels seem related to SUR1 function in the heart and other tissues. An initial hypothesis suggested that differences in SUR1 expression between the heart and brain could be associated with epigenetic regulation. At least for the distal region of the promoter (−517 to −196), we can reject this hypothesis because the results indicated that AciI and BstUI sites in both tissues were not methylated. Consistently, it has been reported that the *Abcc8* promoter sequence (first exon and proximal upstream region −538 bp) in heart and HL-1 cardiomyocytes contains low methylation rates of approximately 0.14% of the CpGs residues (Fatima et al., 2012). Due to methodological issues, we could not amplify the region −51 to +83 bp relative to +1 because of the high content of CGs in this region. Thus, a different methodological strategy should be established to perform a complete analysis of the promoter and, therefore, be able to determine if DNA methylation is a crucial factor contributing to the repression of SUR1 expression in the brain.

The low levels of SUR1 isoforms detected in the brain under physiological conditions increased after 2 h of MCAO followed by 24 h of reperfusion. This effect was reproduced in the cellular line of microendothelial brain cells exposed to OGD followed by a recovery period. Importantly, we observed that the activation of SUR1 induced an increase in intracellular Na^+^ content, an event that is coupled with water movement and edema formation. Overexpression of SUR1 is accompanied by concomitant increases of the TRPM4 and the water channel aquaporin 4, triggering edema formation in brain tissue (Alquisiras-Burgos et al., 2020; Stokum et al., 2023). Therefore, SUR1 increased expression is associated with the biochemical and molecular reactions activated in the brain after an ischemic stroke.

On the contrary, under normal conditions, SUR1 is abundantly expressed in the heart (Hernández-Sánchez et al., 1997; Shi et al., 2005); however, SUR1 expression has not been fully characterized after myocardial pathologies. Myocardial ischemia can lead to atrial fibrillation and cardiac arrhythmias (Staerk et al., 2017). Notably, we found that the abundant expression of SUR1 in the heart was markedly reduced after ischemia/reperfusion. K_ATP_ channels are activated when there are low intracellular oxygen levels and ATP/ADP ratios. Thus, it is assumed that under conditions of acute metabolic impairment, activated K_ATP_ channels can be cardioprotective by shortening the action potential duration, reducing excessive calcium entry, and stabilizing the cardiac membrane potential (Lefer et al., 2009; Flagg et al., 2010). However, reduced availability and functionality of K_ATP_ channels observed after ischemia/reperfusion (due to the diminished expression of SUR1) could make the heart more susceptible to ischemic damage by increasing the risk of arrhythmias and potentially causing an abnormal heart rate (Maslov et al., 2023).

Kir6.2 and SUR1 knockout exert cardioprotective effects, preventing atrial arrhythmogenicity and minimizing alterations in atrial electrophysiological parameters during hypoxia (Specterman et al., 2023). Likewise, Elrod et al. (2008) observed that SUR1-null mice showed a reduced infarct area, suggesting a regulatory mechanism of the SUR1 subunit in the damage induced by myocardial ischemia. These inconsistent results suggest that SUR1 exerts different functions in the atrium and ventricle of the heart.

Different damage mechanisms can be activated during ischemia/reperfusion. For instance, Ca^2+^ transients in cardiomyocytes exposed to OGD increased amplitude, probably because more L-type voltage-dependent Ca^2+^ channels were recruited. Ca^2+^ overload during ischemia/reperfusion may promote the “reverse mode” of the Na^+^/Ca^2+^ exchanger, allowing Ca^2+^ to enter the cell instead of being extruded (Pott et al., 2011). Ca^2+^ overload in cardiomyocytes can disrupt mitochondrial function, activate proteases, increase reactive oxygen species production, and exacerbate the proinflammatory response leading to cell injury and death (Bompotis et al., 2016; Wang et al., 2020).

Diazoxide, an opener of the mitochondrial K_ATP_ channels, may act as a cardioprotective drug by decreasing cell death during ischemia (Liu et al., 1998). Nonetheless, Diazoxide induced noticeable changes in the Ca^2+^ transients of OGD-treated cardiomyocytes, probably because of the decreased expression of SUR1 in these cells. The rise in intracellular calcium induced by OGD likely evokes a rapid and complete inhibition of K_ATP_ channels (Findlay, 1988). Therefore, the loss of SUR1 expression induced by ischemia/reperfusion in the heart might negatively affect K_ATP_ activity and trigger cellular damage.

In conclusion, a comprehensive understanding of SUR1 regulation in the brain and heart could have many promising perspectives. For instance, the short SUR1 isoforms detected could be associated with mitochondrial K_ATP_ channels, whose activity has to be studied. Additionally, the ischemic processes differentially regulate SUR1 expression depending on the tissue injured by ischemia, and the analysis of the regulatory mechanisms of the *Abcc8* expression may provide invaluable information that helps to target SUR1 and reduce cell injury and death in tissues exposed to ischemia.

## Data Availability

The original contributions presented in the study are included in the article/Supplementary material, further inquiries can be directed to the corresponding author.
