# Supplementary material for: Expression of SUR1 isoforms in the brain and heart after ischemia/reperfusion
Source: Front Mol Neurosci. 2025 Apr 17;18:1536409. doi: 10.3389/fnmol.2025.1536409 (PMC12043708; doi:10.3389/fnmol.2025.1536409)
Supplement: Supplementary file 1 [file Table_1.docx]

| **Table supplementary 1. Sequences of PCR primers used to amplify Abcc8 gene exons** | | | | |
| --- | --- | --- | --- | --- |
| **Abcc8 gene**  **Exon** | **SUR1 Domain** | **Fordward primer** | **Reverse primer** | **Product size**  **(base pairs)** |
| E1 and E2 | NH2 | 5´-ATGCCTTTGGCCTTCTGCG-3´ | 5´-ATGTGCACCTTGGAGCTCTG-3´ | 179 |
| E5 and E6 | NH2 y TM0 | 5´-AGAGCCCTCACCAACTACCA-3´ | 5´-GCCAGGATACGGAATGTGCT-3´ | 155 |
| E10 and E11 | Link TM1 | 5´-TGGAGAAGACACGCAGGAAG-3´ | 5´-GATGAGGACAGCAGCGATGG-3´ | 107 |
| E14 and E15 | NBD1 | 5´-GGATGGAGACGCTGACAACT-3 | 5´-GGGGGATACGGATGGTGATG-3´ | 104 |
| E17 and E18 | NBD1 | 5´-TGTGGCAAGTCCTCGCTC-3´ | 5´-TCTTCTCCCTCGCTGTCTGG-3´ | 101 |
| E23 and E24 | NBD1 | 5´-TGGAAGACCCTCATGAACCG-´3 | 5´-CCTCCTCATCCTCATCCAGC-3´ | 139 |
| E35 and E36 | NBD2 | 5´-CTCCGCTCACGCCTGTCTATCAT -3´ | 5´- CCACAGCGTGCTGTCTGAGCAT-3´ | 105 |
| NCBI Sequence: NM_013039.2 *Rattus norvegicus* ATP binding cassette subfamily C member 8 (Abcc8).  Abbreviations: E: exon; NBD, nucleotide binding domain; NH2, amino terminal; SUR, sulfonylurea receptor; TM, transmembrane domain. | | | | |
